# The Medicinal Leech

**Published:** 1826-07

**Authors:** 


1. *A Treatise on the Medicinal Leech, including its Medical and Natural History, with a Description of its Anatomical Structure: also, Remarks upon the Diseases, Preservation, and Management of Leeches.* By JAMES RAWLINSON JOHNSON, M.D. F.L.S, &c. Illustrated with two Engravings. Octavo, pp. 147, London, 1816.

2. *Further Observations on the Medicinal Leech; including a Reprint, from the Philosophical Transactions, of two Memoirs, comprising Observations on the Hirudo Vulgaris, or common Rivulet Leech; and on the H. Stagnalis and H. Complanata, now constituting the Genus Glossopora, with Illustrative Drawings.* By JAMES RAWLINSON JOHNSON, M.D. F.R.S. F.L.S. &c. 8vo. pp. 112, with a Plate. London, December, 1825.

3. *Article SANGSUE.* Par M. MERAT. Dict. des Sciences Medicales. Tom. 49. page 520 *et seq.*

4. *A Treatise on the Utility of Sanguisuction, or Leech-Bleeding, in the Treatment of a Great Variety of Diseases, &c. &c.* By REES PRICE, M.D. Surgeon, &c. 8vo. pp. 152. London, 1822.

THE animal kingdom has furnished us with few medicinal agents of any great importance—the leech and the lytta excepted. Of these two, the former most unquestionably claims precedence, though certainly they are both of the highest utility in therapeutics. The leech is every day rising in importance, as the instrument of local depletion, and must always be considered superior to cupping, on account of its facility of application to parts where cupping-glasses could not be applied. The pressure of the latter instrument is also a decided objection in many cases of disease, especially abdominal inflammations. Indeed, in all superficial inflammations, blood can only be drawn from the neighbourhood of the disease, and not from the immediate seat of it—whereas, the leech may be applied on the inflamed part itself, with very few exceptions. The scarcity of the leech in these Islands, which may partly be traced to the destruction of its accustomed haunts by the cultivation of our waste lands—and partly to the increased employment of the animal, unfortunately renders us dependent on a foreign supply which, in times of war, must always be precarious. Hence, many persons, even above the lower classes, are excluded from the benefits of the animal by the greatness of its price—or purchase them by sacrifices and privations which they can but ill sustain. Every mean, therefore, by which the propagation of the leech can be facilitated, and its mortality checked, deserves to be most attentively explored and sedulously cultivated. It is evident that these objects can only be attained by close observation of its natural habits and propensities, and by correct acquaintance with its structure and physiology. On this account, we shall take a comprehensive view of the subject, and hope, by the extensive diffusion which it will receive through the medium of our journal, on both sides of the Atlantic, to render some service to the profession at large. We shall follow Dr. Johnson's arrangement, adopted in his first work, as best calculated to forward our objects in this eclectic article.

Section I. *The Medical History of the Leech.* Casual observation of the effects of the leech, in its attacks upon other animals, probably suggested the first idea of its introduction into medical practice; and experience of its beneficial operation on the human frame, when suffering from accident or disease, would alone be wanting to sanction and extend its employment. During the infancy of medicine, in the schools of Egypt and of Greece, the uses of the leech appear to have been unknown. Themison, disciple of Asclepiades, and founder of the methodic sect, who practised at Rome about the close of the reign of Augustus, or commencement of that of Tiberius, first notices them in his writings. He applied a cupping-glass over the orifice, formed by the leech, in order to augment the flow of blood. Of his success in the practice, nothing can be learned. Among the more celebrated authors, who subsequently wrote in commendation of the virtues of the leech, may be principally enumerated, Pliny the naturalist, Aretæus, Ætius, Ægineta, and Zacutus Lusitanus. By these it was, for the most part, employed, or recommended, against topical pains and inflammation. In several forms of chronic and febrile disease, abstraction of blood, by its means, from the hæmorrhoidal veins, was deemed an almost unfailing remedy. If, in the present day, the intimate connexion which subsists between those veins and the system of vessels constituting the portal vein and inferior cava, were better recollected, and the indications which it might suggest in diseases of the abdominal organs, more frequently acted upon, the reputation of our science and the interests of the sick would, we are convinced, be, on many occasions, very materially promoted. Much too as the practice of the French physicians in this respect has been ridiculed and decried by some of our countrymen, it is equally certain, that applications of the leech to the external sexual organs of the human female, constitutes one of the most direct and unerring means whereby the sufferings to which she is exposed, from frequent disturbance of the functions of the uterine system, may be relieved. Zacutus records a case of phrenitis, consequent on retention of the menstrual flux; wherein, after the failure of the wonted remedies, the application of four leeches, properly secured by thread, to the vagina in the immediate vicinity of the orifice of the womb was followed by cessation of the phrenitic symptoms and speedy convalescence. Nor, in the inflammations, chronic or acute, of the serous membranes lining the great cavities, is the decided superiority of sanguineous depletion by leeches, from the neighbourhood of the inflamed membrane, to the practice of venæsection, generally understood or sufficiently appreciated. Little difficulty would be encountered in tracing the medicinal employment of the leech down to the present day, but enough has been advanced to demonstrate its antiquity; and the evidence of succeeding ages has served only to exalt the character of the remedy, and fix it upon a surer basis.

Let us turn rather to contemplate the formidable accidents which may result from its employment, and consider the most prompt and effectual means of obviating the dangerous consequences sometimes induced by fortuitous introduction of the leech into the fauces, or intestinal canal, of the human subject. Such accidents have not unfrequently been witnessed; and in the writings of the ancients may be found a description of the symptoms denoting the presence of the leech in the throat or stomach, and an enumeration of the remedies best calculated to dislodge the terrible intruder.* Of the latter, the principal are vinegar, garlic, salt water, assafœtida, mustard, and other acrid substances, copiously administered; sternutatories of elaterium and hellebore; or, when pain in the throat indicated that the animal was there, retention of cold water in the cavity of the mouth, whereby it might be decoyed into a situation less inaccessible and remote. But the celebrated French surgeon, Larrey, of all the authors with which we are acquainted, has

* Pliny says that the elephants which happened to swallow leeches while drinking, were cruelly tormented by these animals in their stomachs. "Cruceatum in potu maximum sentiunt haustâ hirudine, quam sanguisugam vulgo cæpisse appellari adverto." *Lib.* viii.

given the best description of the phenomena resulting from the attachment of a peculiar kind of Egyptian leech to the faucial membrane, and of the method of treatment, by which the dangers of the accident may be averted. The introduction, therefore, of a brief abstract from the Baron's interesting memoirs,* will not be considered, by the reader, irrelavent to our subject, or destitute of instruction.

During the operations of the French army in Egypt, pools of soft and muddy water were frequently met with, particularly in the deserts bordering upon Syria. These contained, among other animals, a species of leech, which, though but a few millimetres in length, and fine as a horse-hair, acquired, when gorged with blood, the volume of an ordinary leech. It was black, and somewhat resembled the species which has been observed in the island of Ceylon. The soldiers, urged by thirst, and unsuspicious of danger, threw themselves down upon the margins of these lakes, and drank with avidity; but many soon experienced the baneful effects of the leeches which they had swallowed. Painful sense of stinging in the posterior fauces, frequent cough and expectoration of glairy mucus streaked with blood, and disposition to vomit, were the primary consequences of the bite. To these symptoms succeeded swelling of the throat; frequent hæmorrhages; difficulty of swallowing and respiration; pains in the breast; increased cough from the irritation excited by the tail of the animal on the borders of the glottis, and perhaps also by that of the effused blood upon its orifice; perceptible emaciation; loss of sleep and appetite; restlessness and agitation, sometimes, unless timely assistance were afforded, terminating in death. On examination of the throat of the first individual attacked by these symptoms, a leech, of the size of the little finger, was discovered in the isthmus of the fauces, and with difficulty extracted by the polypus forceps. Slight hæmorrhage succeeded the operation; and the patient soon recovered. In other instances, gargles of vinegar and salt water, injections of salt water, fumigations of tobacco and squill, were successfully employed to dislodge the leech from the posterior fauces. Sometimes the animal descended into the œsophagus or stomach, from whence, after remaining some time, it was, at last, detached by the use of diluted vinegar, or by the contraction of the viscus itself. M. Latour-Maubourg, chef de brigade, in drinking the water of a small lake on the deserts of St. Makaire, about one day's jour-

---

* *Memoires de Chirurgie Militaire, &c.* Tome i. page 359.—See also the translation published by Mr. Waller.

ney from the pyramids, swallowed two of these leeches; by which he was dreadfully harrassed during the rest of his march, and reduced to the last extremity of weakness and exhaustion. The cough and bloody expectoration continued after his arrival at Cairo; and medicine, their origin not being suspected, served but to aggravate his symptoms. At length, the leeches were discovered; one of them extracted by the forceps from the pharynx, and the other dislodged from the nasal cavities by salt-water injections. A leech, situated like this last, in another subject, was only distinguished from nasal polypus by its sudden retraction on the contact of the instrument employed by M. Larrey to detach it. Persons, who, in traversing these deserts, are under the necessity of drinking water infested by such animals, should previously pass it through strong linen; and, if possible, add to it a few drops of some kind of acid. Should a leech penetrate into the human stomach, it is highly probable, he thinks, that it could not long exist there; although the facts, observed by Dr. Johnson, warrant an inference, that the animal, until its vital principle became extinct, would resist the solvent action of the gastric fluid. Under these circumstances, copious ingestion of salt water, would, doubtless, accelerate its destruction.

To this account, Dr. Johnson, in his last work, has added an extract from a paper entitled "*Observationes de Cardialgia Hirudinosa*," in which is reported the case of an individual, who, having for some time laboured under a cutaneous affection, was advised to try the sulphureous waters of Baden. In his journey thither he drank some muddy water, shortly after which, he felt pain in his stomach, which continued nearly six months, being sometimes so violent as to throw him into convulsions. He took a variety of medicines, without effect. At length, after taking an emetic, he brought off five leeches—one of them nearly the size of his finger, the rest comparatively small, but all turgid with blood. From this moment he was freed from his complaint.*

In this place it may not be improper to advert to the leech of Ceylon, which proves so incessant and tormenting a plague to travellers in that Island—as reported by Dr. Davy and others. The largest of these animals are seldom more than half an inch in length—broad behind and tapering before—and in substance semi-transparent. It is very active, and is supposed to have an acute sense of smell, for no sooner does a person stop where leeches abound than they crowd eagerly to the spot from all

* Ephemerides des Curieux, &c. 1719.

quarters. In rainy weather it is shocking to see the legs of men on a long march, thickly beset with these creatures gorged with blood, which is also trickling down in numerous streams. It is extremely difficult, if not impossible, to ward off their attacks. Their bites too, are exceedingly troublesome, being apt to fester and become ulcers, sometimes occasioning the loss of a limb, or even of life. The instant a leech fastens on, an acute pain is generally felt, like that produced by the bite of the medicinal leech; and, in a few hours afterwards, the surrounding skin becomes inflamed, with most troublesome itching. This itching sometimes continues several days, till the wound is either healed or ulcerates.

The ancients, whenever the hæmorrhage, consequent on application of leeches, was unnecessarily protracted, relied for its suppression, on plugging the orifice with powdered aloes, bole armeniac, or sponge dipped in liquid pitch; but continued pressure with the finger, whenever the orifice is situated over a bone, will be found a much more direct and efficient mode of accomplishing this object; or, under other circumstances, especially if the bleeding prove very obstinate and menace dangerous consequences, the formation of an eschar by the employment of caustic may be had recourse to with almost invariable success. The method in vogue now is, the passing of a common sewing needle across the wound, letting it remain there and twisting it round, so as completely to prevent the egress of blood. The idea that forcible extraction of the leech from its hold could not be safely attempted, is completely disproved by the unceremonious practice of the French army surgeon; and hence, the story of the death of the Roman Consul, Messalinus, in consequence of the teeth of a leech being left in the wound which it had inflicted, is probably incorrect.

SECTION II. *The Natural History of the Leech.* Under the genus *Hirudo*, belonging to the order of *Vermes externi* in the Linnæan arrangement, many species are comprehended; but much confusion has arisen from the introduction into it of several individuals of other families, differing essentially from the generic character, as delineated by the Swedish Naturalist.*

---

* The *Hirudo viridis*, of Dr. Shaw, the *H. alba* and *H. nigra*, of Mr. Kirby are now included in the genus *Planaria*. Dr. Johnson also constitutes a new genus, which, from the retractile tubular tongue of the animals composing it, is entitled *Glossiphonia* (γλωσσα, a *tongue*, and σιφων, a *tube*. The following is the generic character which he delivers: Corpus subovatum depressum, caput acuminatum, lingua tubulata, os cau-

*Hirudo. Character Generis.* Corpus oblongum subrotundum, anterius et posterius truncatum, muticum, cartilagineum, os caudamque dilatando progrediens. The genus may be divided into those which occupy rivers or stagnant waters, and those which inhabit the ocean.—To the three first species of the former division, *Hirudo Medicinalis, Sanguisuga,* and *Troctina,* our observations will be principally restricted.*

*H. Medicinalis.* Hirudo depressa nigricans, supra lineis flavis sex, intermediis nigro arcuatis, subtus cinerea nigro maculata. Oculi decem, more delineato. Long. Pollices tres. In stagnis—paludibus. Caput, quiescens, subrotundum, progrediens accuminatum. Os, quoad figuram mutabile, rimam triangularem plerumque exhibens. *Cauda,* circularis, complanata, fibris carnosis e puncto centrali divaricatis.

*H. Sanguisuga.* Hirudo elongata fusco-viridis, subtus cinereo virens, maculis nigris. Oculi decem. Long. Pollices tres. In stagnis—locisque palustribus.

*H. Troctina.* Hirudo elongata fusca, supra annulis aureis, maculas atras cingentibus, margine sub-flavo laterali, subtus flavo-viridis punctis atris. Oculi decem. Long. Pollices tres. In rivis—Piscibus crebro adhærens.†

---

damque alternè affigendo progrediens. Under this genus, he arranges the *Hirudo complanata* and *stagnalis,* by the respective titles of *G. Tuberculata* and *G. Perata.* He thinks also, that the *Hirudo Crenata,* of Mr. Kirby, and the *H. circulans,* inhabiting the Thames, will be ultimately found to belong to this genus. Indeed, it appears probable, that the *H. Crenata,* though described by Mr. Kirby as possessing only *two* eyes, is identically the same as the ordinary *H. Complanata:* for, singularly enough, the figure which accompanies Mr. Kirby's description, is delineated with *six* eyes, and has, moreover, a *retractile tongue.* Such is the argument of Dr. Johnson. We have not paid to this subject attention sufficiently minute to render decisive our opinion upon the justice of his ideas, and the correctness of his classification.

* The remaining species of the *fresh-water* division here enumerated, are the *H. Nigra, H. Vulgaris, H. Tessulata, H. Lineata, H. Heteroclyta, H. Geometra,* and *H. Marginata.*—*H. Indica, H. Grossa, H. Hippoglossi, H. Branchiata, H. Muricata, and H. Verrucosa,* are the species found in the *ocean.*

† "I am not aware," says Dr. Johnson, "of this species having been hitherto described. I have denominated it *Hirudo Troctina,* from its resemblance, in regard to the coloured rings or spots, to the trout, and also from its being known and sold in the shops under the name of the Trout-leech. Owing to the great scarcity of the medicinal or striped leech, it has been latterly employed in medicine."

*Appellations of the Leech in various Countries.* By the Greeks it was named βδελλα; by the ancient Romans, *Hirudo* (from *haurio*, to draw out); by the moderns, *Sanguisuga*. The French call it *Sangsue;* the Italians, *Sanguisuca;* the Germans, *Blutegel;* those of the Low Countries, *Lyche lake;* the Danes, *Blodigle;* and Poles, *Pijavvka*.

The medicinal Leech is common throughout Europe, more especially in the southern parts. It is often seen, in the south of India and America, to measure six or seven inches, twice the common length of the European Leech. The predominant colour of the animal varies according to the colour of the soil which forms the boundary of its habitation; but the spots or lines with which it is marked, are nevertheless permanent; and hence invariably denote the specific character. Of the motions and mode of progression of leeches, we need not pause to speak. During winter, they inhabit the deeper waters; during summer, the shallows. If the cold of the former season be very rigorous, or the drought of the latter excessive, they retire deep within the ground, leaving a small aperture to their subterraneous abode. They commonly re-appear about the close of March. In cold and cloudy weather, they do not quit the mud. Before a thunder-storm, they rise to the surface, and may be collected with facility. Though considerably affected by atmospherical vicissitudes, they certainly do not predict, with any thing like precision, the changes of the weather. Vigilant observation, made for some weeks on a vessel containing a number of leeches, will suffice to convice any one of the correctness of this assertion.*

The fluids of fish and frogs constitute the food of the *H. Medicinalis* and *Troctina;* but neither earth-worms, nor the larvæ of aquatic insects, are attacked by them. The *H. Sanguisuga*, on the other hand, possesses a voracious appetite, and the faculty of ingesting solid aliment. When urged by hunger, it will seize on the medicinal leech, and even devour individuals of its own species. Dr. Johnson saw one of these gluttonous animals swallow whole, within a very short time, two of the *H. vulgares*, or rivulet leeches; and, three days afterwards, one of them was rejected "in a *living* state," apparently having suffered but little injury from this awkward discipline;

* It has been stated, on the authority of a French clergyman, that leeches predict changes in the weather with great accuracy. And our own poet, Cowper, was of the same opinion. "Leeches, says he, in point of the earliest intelligence, are worth all the barometers in the world." This, we think, is a great exaggeration. We never could discover any movements in the leech that could be considered as predictive of atmospherical changes.

a few hours only elapsed ere it was again swallowed. Another of the *H. Vulgares* was observed to deposit several ova, at distinct intervals, after liberation from two hours' confinement in the intestinal canal of the *H. Sanguisuga*. Dr. Johnson also found, from constantly supplying two of these animals with the *H. Vulgares*, both living and dead, that, within thirty days, one of the former devoured fifteen, and the other, twenty of the latter. During the whole period, the water, though occasionally renewed, was turbid, from the copious evacuation of thread-like fæcal matter. The digestive powers of the *H. Sanguisuga* appear to possess considerable vigour and activity; for, upon opening two of them, which had each, five days previously, swallowed a rivulet-leech, not a vestige of it could be discovered in the alimentary canal. This peculiarity constitutes a striking exception to the general law of tardy digestion in cold-blooded animals; and is elucidated by a view of the peculiarities of anatomical structure which distinguish the horse-leech: for the intestine of this species is more than double the width of the same organ in the Medicinal and Trout-leech; nor is the stomach so thickly set with membranous folds or partitions. Analogy renders it probable, that the digestive organs of this voracious animal act with increased energy during the summer; and it evidently possesses the power, enjoyed by carnivorous birds, of rejecting, at pleasure, recently swallowed food.

The result of Dr. Johnson's numerous observations and experiments upon the two respective species of this very singular genus is, that the *Medicinal Leech* takes no solid aliment; that, in its native abode, it subsists on the fluids of fish, frogs, &c.; that it possesses not the voracity which distinguishes the Horse-leech; and that it betrays no propensity, like the latter, to destroy its own species, or any other belonging to the same genus.* With respect to the *Horse-Leech*, the fact is, that it

---

* Plenipus remarks that he has known leeches exist three years in water without any other nourishment. But it is to be remembered that the stomach of the leech will contain from half an ounce to an ounce of blood, and that this fluid is received into several cells or partitions, over which the leech has perfect control, merely allowing so much nutriment to pass into the alimentary canal, as is sufficient to preserve its existence. This may diminish our surprise at the long abstinence of the animal. In short, its stomach may be regarded as a general store-house, upon which it can draw for food as often as its necessities require. It, therefore, carries within itself a sufficient supply for several years, and only requires renewal of the water, from time to time. Upon examination, it has been found that the blood, after remaining some months in their stomach, undergoes but little change, either in colour or fluidity—at least in the first eight cells.

devours alike the *H. Medicinalis, H. Vulgaris,* and even the weaker of its own species, in the absence of other alimentary substance; and will swallow almost any thing within its reach. Hence the designation of *H. Vorax* is more characteristic of, and correctly applicable to, the species, than that of *H. Sanguisuga.*

Leeches have been said to possess the extraordinary property of regeneration, when mutilated, or even divided into several portions. The experiments of Dr. Johnson render it probable that this statement is incorrect.*

The leech is exceedingly tenacious of life. Not only will it exist, when deprived of its head or otherwise mutilated, for several months, but sustain little apparent injury or inconvenience from confinement, of many days, beneath the exhausted receiver of an air-pump. A leech thus circumstanced, has been known to support the state of insulation more than three weeks. It is, moreover, demonstrated by experiment, that the animal will live in a glass vessel, containing three cubic inches of hydrogen gas, 2 days 12 hours; a like quantity of carbonic acid gas, 5 hours; of nitrogen gas, 8 days; of atmospheric air, 10 days; oxygen, 12 days; water, impregnated with carbonic acid gas, 4 hours; olive oil, 1 day 16 hours; spring water, the vessel being stopped, 7 days. The organs of the young leech are exceedingly tardy in their development. About five years are supposed to elapse ere they attain a state of maturity. It is probable that the animal will live in its native waters, with an abundant supply of food, at least twenty years.

Section III. *The Anatomical Structure of the Leech.*†

We have now to contemplate the Leech, first, in its *external structure;* secondly, in the constitution of its *sentient and respiratory organs;* and, thirdly, its *internal structure.* For the sake of brevity, we shall endeavour to introduce into this part

---

* According to Vitet, if we take a leech, and cut off both its anterior and posterior extremity—taking care to renew the water every second or third day—it will, thus mutilated, live two years or more, apparently unaffected in strength or activity. But the amputated parts are never regenerated. The general distribution of the nervous system, in this animal, which has no common centre of life, accounts for its tenacity of vitality, when mutilated. Vitet ascertained that the leech dies almost immediately when immersed in oxygenated muriatic acid gas, and that the dead animal will remain several years in a vessel filled with this gas, without undergoing any decomposition. Hence, he thinks, this gas may be employed with great success in the preservation of dead animals, particularly insects.

† Two engravings, as correct as neatly executed, illustrate this description.

of our article the concise and rigorous character of botanical description.

*External Structure. Body*, when quiescent, arched, and in length more than an inch; capable, when moving, of great extension; furnished with rings or annular muscles, usually about one hundred, increasing in size, not in number, with age, and each presenting, on its most prominent part, a row of minute tubercles. Mouth, of a circular or horse-shoe figure, but capable of assuming any other; external surface dark grey; internal, light grey; upper lip slightly bent downward with a central cleft; lower, bent inward, and, like its fellow, semicircular. Sucker, at the oral extremity,* formed by fasciculi of muscular fibres, some disposed in a radiated, others in a circular, direction. Caudal sucker, similarly constituted. First generative orifice, situated in the abdomen, half an inch from the lower lip, round, small, affording passage to the male organ of generation:† second orifice, or vagina, leading to the uterus, distant about five rings from the former, towards the caudal extremity, and with difficulty perceptible: third orifice, or anus,‡ placed on the back, very near the rim of the caudal sucker, and capable of admitting a pin, although smaller in the *H. Medicinalis* and *Troctina*, than in the *H. Sanguisuga.*

ORGANS OF THE SENSES. *Organ of vision.* Eyes ten in number;§ arranged in a crescent shape, at the pointed extremity on the back of the head, the two at each extremity of the arch, more distant from each other than the rest; deep black; when moistened, possessing a fine lustre, and seen beneath the microscope as tubercles jutting from the skin. *Organ of touch*, residing in the lips, and perhaps in the disk which terminates the caudal extremity. *Of taste*, composed

---

* We have taken the liberty of substituting the more precise terms of *oral* and *caudal* sucker, for superior and inferior. They constitute the two extremities of the animal; and by their means it is capable of very firmly attaching itself to any surface.

† This has been mistaken for a respiratory orifice; but the penis may frequently be seen protruding from it, particularly in the dead leech.

‡ By many celebrated naturalists, the leech has been described as destitute of an anus; but the inaccuracy of this statement is proved by the fact, that a fluid may be so injected through the mouth of the animal as to issue in a stream from the dorsal orifice, and vice versâ. Fæcal matter may also, by compression of the body of the leech, be forced out of this opening.

§ This description applies only to the *H. Medicinalis, Sanguisuga, Troctina*, and *Nigra.* They "who wish to have preparations of these organs, may readily form them, by cutting off the head of the leech, and subjecting it, between two strong plates of glass, to continued pressure."—Dr. JOHNSON.

of an assemblage of nervous fibrillæ situated in the upper part of the œsophagus. *Of hearing*, none yet discovered. *Of smell*, probably will be found to exist in the puncta respiratoria, by which the leech is very confidently believed to breathe.

*Respiratory Organs.* The opinion of naturalists on this subject is much divided. Some contend that the leech respires by the mouth; others, among whom is Dr. Johnson, that the process is executed by means of spiracula or breathing-holes.*

*Internal Structure.* An epidermis, cutis, muscular and membranous coats, with the interposed cellular substance, constitute the coverings of the leech. *Epidermis*, or outer membrane, thin, fibrous, reticulated; cast off every fourth or fifth day, and seen, like a small ring, floating in the water. *Cutis*, externally dense and firm; internal surface flocculent; texture spongy; firmly attached to the subjacent muscular coat, especially in the inter-annular spaces, where much of the flocculence disappears. *Muscular coat*, ash grey, strong, elastic, consisting of two strata of fibres, circularly and longitudinally disposed, interlaced and inseparable without injury; the longitudinal very conspicuous. *Membranous coat*, delicate, lining the whole internal cavity; exhibiting, beneath the microscope, the appearance of fine lace.

On dividing the belly of the leech, in a direct line from mouth to anus, but not so deeply as to implicate the alimentary canal, the following organs will be exposed to view: *Three piercers*, incorrectly named teeth, cartilaginous, rounded; furnished with sharp cutting edges; resting on small eminences, and so relatively placed as to meet, under equal angles, in a centre; confined by a strong circular ligament, which surrounds the œsophagus; possessing, when in action, an oscillatory movement, and inflicting a triangular wound.† *A nervous*

* Dr. Johnson is now, however, inclined to admit with Sir Everard Home and Kurzman, that the function of respiration is carried on by what, in his first work, he denominates the *lateral vesicles*. They are sixteen on each side, situated beneath the intestinal canal, containing a quantity of a white fluid which is miscible with water. As the leech then breathes in the same manner as fish, their respiratory organs deserve the name of gill-bags rather than tracheæ.

† Some surprise has been manifested that the leech should be able to inflict a wound with such instruments, which, being entirely cartilaginous, seem little fitted for this office. The wound has, therefore, been attributed to suction alone. But if we reflect on the size of the muscles which regulate the movements of the teeth—their sharp cutting edge—their continual vibration both in effecting the first incision, and in afterwards clearing the passage of any coagulum—and, lastly, the time which the animal takes in making the incision, our wonder will cease.

*mass or brain*, consisting of a ganglion, which surrounds the œsophagus, and sends off filaments to various parts, particularly two lateral nerves proceeding to the tail, and here and there expanding in their course: and a central nerve, occupying the *interior* of the abdominal blood-vessel, and forming diamond-shaped ganglia, which correspond to a similar formation in the abdominal vessel.* *The blood-vessels:* An abdominal blood-vessel, pursuing a direct line from mouth to tail, enclosing the central nerve, and presenting in its course several diamond-like expansions; the first of these observed just below the lip; the second, third, and fourth, respectively, at the origin, middle, and termination of the œsophagus; the fifth, on the bag of the male generative organ; the others equi-distant, and ending close to the disk of the caudal extremity: two lateral vessels, nearly straight when the leech is in motion; when quiescent, assuming an appearance of a circle of festoons; and, lastly, a dorsal vessel, seated on the back, and passing directly from head to tail. All these contain red blood, and communicate by numerous transverse branches, of irregular distribution.† *The male generative organ*, a strong elastic tube, extremely irritable, enclosed in a circular bag, flattened above and below, about one inch in length, often seen hanging from the first generative orifice. *The testes*, oblong bodies, containing a thick greyish white fluid, placed at the commencement of the first pair of cells or stomachs, on each side of the male organ, and transmitting by tubes their contained fluid to the generative bag.‡ Several oval bodies *(the abdominal vesicles,)* of the size and colour of mustard-seed, placed in pairs on the surface of the cells or stomachs (the first pair near the testes, the others equidistant and ending about the middle of the last stomach,) connected to the testes by a tortuous tube, and containing a similar fluid. *The female generative organ*, an oblong sac, somewhat resembling in figure a bagpipe, composed of a strong membrane covered with muscular fibres; connected with the vagina, the orifice of which has been described; and furnished posteriorly with an oviduct leading to the ovaries: it is en-

* By Hunter, Poupart, and Cuvier, the abdominal blood-vessel, presently to be described, has been erroneously represented as the main organ of the nervous system.

† A distinct systole and diastole is perceptible in these vessels on opening the *H. Medicinalis alive*, or holding up to the light the *H. Vulgaris*. In the former, Dr. Johnson has counted ten; in the latter, eight of these pulsations in the minute.

‡ Some writers have regarded these as a nervous mass or brain, and the bag of the male generative organ as an uterus.

dowed with a strong peristaltic motion. *The lateral vesicles*, membranous sacs, about thirty in number, occupying the site of every fifth ring, on the sides of the body, and containing the unctuous fluid for lubrication of the surface. *The œsophagus*, about one fourth of an inch long; at first narrow, gradually widening, and again contracting as it reaches the first pair of gastric cells; furnished internally with several longitudinal muscular folds, whereby the motions of the piercers are regulated, externally, with several strong muscular bands: *The cells*, twenty-two in number, or rather a *single stomach* divided into so many partitions; formed by reflection of the internal membrane, and constituting four-fifths of the bulk of the animal; semi-oval in figure, except the two last, which are oblong and terminate in blind sacs close to the anal extremity.* *The alimentary canal*, a continuation of the œsophagus, about the size of a crow-quill; furnished with openings on each side, which lead, and correspond to the several cells, and throughout with membranous folds, which perform the office of valves in preventing a return of the alimentary matter: And, lastly, *the intestine*, about an inch in length; situated between the two last long cells, and confined by two ligamentous bands obliquely directed at its upper part; furnished at the gastric orifice with a valve which precludes regurgitation of the fæcal matter, commonly filling it, into the stomach; at the anal extremity, with a sphincter which opposes the escape of blood per anum in an overgorged leech. The diameter of this canal is small in the *H. Medicinalis*.

Section IV. *The Diseases, Preservation, and Management of the Leech.* The leech is said, like other animals, to be infested with lice: and from the frequent occurrence of the following forms of disease, great mortality prevails among the species: 1. An ulcer, variously seated, commonly on the side, presenting at first, only a small ulcerous speck, afterwards spreading in a very rapid and malignant manner; sometimes superficial, sometimes burrowing internally; often tinged with blood; very destructive: the part, constituting its seat, usually contracted.† 2. Another affection, equally malignant,

* The remarkable structure of the stomach may be advantageously exhibited by immersing a leech, when fully gorged with blood, for a week or ten days, in a saturated solution of muriate of quick-silver, and afterwards opening it and picking out with a needle the contents of each cell: or, by filling the cells of the recent dead animal with crude mercury, the precise figure of each will be more clearly demonstrated.

† This ulceration is particularly common in the *H. Vulgaris*.

wherein one portion of the body is contracted in diameter and rigid, and another studded with tumors presenting, on incision, black putrid, coagulated blood: 3. A general flaccidity of the body, with exception of the lips, which are indurated, swollen, purple, often bloody. These diseases do not exclusively affect the leech in a state of confinement; they prevail particularly during summer; are contagious, and invariably fatal. Hence, any leech, exhibiting signs of indisposition, should be immediately secluded from the rest. Should the water, containing the leeches, display a bloody tinge, an impending mortality is denoted; and it will be prudent to separate, and keep them in small numbers, even although apparently healthy.

Leeches, when in considerable numbers, should be *kept* in a large stone vessel furnished with a false bottom, so perforated as to allow the passage of the animals, and with a stop-cock whereby the water may be drawn off. The false bottom should be so elevated, as to allow the introduction of a turf, or quantity of marsh horse-tail *(equisetum pallustre)* between it and the real one.* The water should be preserved of a regular temperature; changed about once a week; and the situation, chosen for the jar, be free from all unpleasant smell. Previously to introduction, each leech should be narrowly inspected; and all those, whose body feels flabby, or exhibits protuberances or white superficial ulcerated specks, should be kept separate, and have the turf and water of the containing jar frequently renewed.

At the time of Dr. Johnson's first Essay, the natural history of the leech was involved in much obscurity, which has since been cleared up by some Continental writers, especially Dr. Noble of Versailles. This gentleman's essay forms a prominent feature in the second work, at the head of this article, and we shall submit a rapid outline of it to our readers.

Dr. N. believes the leech to be *oviparous*, although the majority of naturalists, have considered it to be *viviparous*—a discrepancy, however, which Dr. Johnson endeavours to reconcile in the course of the work. Dr. Noble observes, that the difficulty of procuring leeches in winter has long induced the leech-dealers to collect them in the autumn, and keep them in vessels for ordinary consumption. But although the water be frequently renewed, great loss is sustained, chiefly by the com-

---

* By these means, the leech is enabled to rid itself of the epidermis, which, unduly retained, might prove a source of disease. During the early part of spring, leech-catchers employ the *equisetum pallustre*, in a dry state, to allure the leeches to the surface of the water.

bats of the little animals, the weaker falling a prey to the stronger, by whom they are devoured. With the view of preventing these accidents, Dr. N. applied to the matron of the Royal Hospital of Versailles, who caused a reservoir to be constructed, seven feet in length by three in breadth and the same in depth, having a southern aspect, and being supplied with a current of fresh water. Its sides were sloping and lined with slime or mud. The borders of this little lake were surrounded with turf and a few rushes, to enable the inhabitants to retire to a shady spot during the summer heats. In November, 1820, they placed a colony of 2000 leeches in this reservoir—the grey and the green leech—and here they passed a severe winter without any sensible loss. When the cold became considerable, they buried themselves in the mud—reappearing only when the atmosphere was warmed by the sun's rays. About the end of spring they began to notice some young leeches adhering to the back and belly of the parents—occasionally swimming about, to try their strength. In the month of August they observed conical-shaped excavations in the slime on the banks, in each of which was found a small cocoon, resembling that of the silk-worm, and about the density of a piece of fine sponge. Several were collected and put into a bottle of water. On opening them, some were found with a vacant cavity, having an aperture at each end—others, of smaller size, were filled with a jelly-like substance—in others still, they found from nine to fourteen young leeches in different stages of growth—this growth appearing to bear a proportion to the development of the tissues forming the body of the cocoon. In a few days the young leeches were seen issuing from the cocoons in the bottle, and swimming freely in the water. On withdrawing the empty cocoons, an aperture was seen at each extremity, through which a pin could hardly be thrust. The internal surface of the cocoon was covered with a dense, smooth, albuminous matter. In the ensuing September, Dr. Noble and others had various opportunities of seeing the leeches actually proceed from the apertures in the cocoons. Dr. N. never could observe the old leech fabricating its cocoon around the albuminous matter, on account of the muddiness of the water after the animal had entered the holes in the banks.

It is highly necessary that the edges of the reservoirs should be well furnished with clay, as a nidus for the leeches in cold weather. Dr. N. here remarks the curious fact, that leeches have the means of securing the product of their fœcundation from injury, by a protecting envelope—a tissue or kind of felt, resembling fine sponge, and probably formed of the mucus or slime, with which their bodies are so amply supplied—especially at certain times.

When this memoir was read at the Society of Agriculture, M. De Planey observed, that the existence of cocoons was well known to the leech-dealers in Bretagne, who by means of them replenished their ponds for the metropolitan market. About the month of April or May, they send out labourers, provided with spades and baskets, to the little muddy marshes, where they collect a quantity of mud known to contain cocoons, which mud is afterwards deposited in sheets of water prepared for its reception. Here the young leeches are allowed to quit the cocoons, and after an interval of six months they are conveyed to larger ponds. Horses and cows are driven into these ponds, with the view of affording the leeches some exercise in their future avocation, and in twelve months the dealers begin to collect them for medicinal use. It appears that these animals do not multiply in great abundance, unless they have tasted blood—particularly that of cows.

From these statements, Dr. Johnson admits that it is satisfactorily proved that the leech is *oviparous*—that it produces a cocoon, like that of the silk-worm, in which are imbedded, in a gelatinous mass, the ova of the future leech. Yet our author adheres to his former opinion that the animal is, *under particular circumstances*, viviparous. The reasons adduced are chiefly analogical; but we do not deem the subject of sufficient consequence to spend any time in its discussion.

In reference to the great mortality produced by the combats of these animals, Dr. Johnson thinks Dr. Noble is in error, as far as respects the medicinal leech. For although they are fond of red blood, they do not suck it indiscriminately. That of their own species is unheeded by them. Our author has never known them attack any of their own species. M. Vitet, who paid great attention to leeches for forty years, is of the same opinion. The propensity to combativeness, however, is pretty strong in the horse-leech. This creature will not only destroy the medicinal leech, but even the weaker of its own species—in this instance, almost rivalling man himself.

In this work, Dr. Johnson has republished, from the Philosophical Transactions, two memoirs—one on the mode of propagation of the Hirudo Vulgaris, or rivulet leech—the other, being observations on the Hirudo Complanata, and Hirudo Stagnalis. These our limits will not permit us to notice. An appendix of *addenda* to the medicinal history, natural history, and anatomical structure of the leech concludes the work. These addenda we have endeavoured to incorporate with the review of his first work, under their respective heads.

We have now to notice some points relative to the *medicinal use of leeching*, which is the subject of the last work on our list—that of Dr. Price. This gentleman has put forth an unostentatious and useful little volume, which we consider it our duty to recommend to our brethren. In noticing the various diseases in which LEECHING is useful, Dr. P. has taken occasion to introduce many practical remarks of much interest, though in an incidental manner. His little work indicates no inconsiderable research among ancient authors—and, what is perhaps of equal value, good sense and judgment respecting those things which pass under his own observation. The first page of the book contains an epitome of the whole, and is expressed in much better language than we are capable of putting together.

"It may, however, be premised, as a general rule, that in all cases of local congestion, and febrile affections, accompanied with increased excitement of any particular organ or part, the abstraction of blood by leeches, immediately from the seat of the disease, or as nearly so as possible, tends to unload the blood-vessels, in a state of congestion, and to allay morbid action, in a more direct manner than can be accomplished by any other method of abstracting blood—particularly in those cases where, either from the peculiarity or state of the constitution, from age, sex, or a variety of other causes, general blood-letting is inadmissible; the application of leeches, in such cases, is our sheet-anchor, not to be supplied by any other remedy.

"But it must here be noticed, that as leeches principally remove blood from the capillary system of vessels, the effect of the sanguineous discharge is not speedy enough upon the constitution in general; nor does it sufficiently unload the part itself, as to be trusted to without the aid of phlebotomy, where disease has attacked an internal structure or organ, immediately connected with the vital function of the body.

"In acute inflammation, then, of the contents of the chest, abdomen, and of the head, or high inflammatory and febrile excitement of the general system, a prompt and copious blood-letting, by the lancet, should first check the violence of the malady, when leeches may afterwards keep down the disease, and conduct it to a favourable termination, without occasioning that expenditure of strength, produced by the loss of considerable quantities of blood from the larger venal branches." 8.

This short passage contains every thing that is necessary to be said on the subject. Whether the inflammation be internal or external, the propriety and the utility of leeching are unquestionable. If the inflammation be seated in an internal organ, the *local* must, of course, be aided by *general* bleeding and other antiphlogistic means, in proportion to the violence of the disease. And, in truth, the same may be said of an external inflammation.

But there is some management necessary in the application of leeches, which is too often thought beneath the notice of the physician and surgeon. The part, for instance, should be shaven, if covered with hair, and washed clean with *plain* soap and water—then with water alone—and lastly with milk—or what is better, with porter. The leeches themselves should be taken out of the water in which they are kept, and placed, for a few minutes, on a dry cloth, in order to clear themselves of the slime with which they are bedewed. They are then to be put into a cup of porter, which will induce them to bite with great avidity. In some countries porter is not a very common commodity, and then, if the leeches do not quickly take, some feathers are to be plucked from the wing of any bird, and the points being snipped off, are to be applied to the parts where the leeches are intended to fix. The consequence will be, that the little animals will greedily bite on the spot where the fluid from the feather is deposited.

The leeches are to be placed in a wine glass or tumbler, with a piece of stiff paper to prevent some of them from clinging to the bottom, and also for them to fix their caudal suckers to, when the glass is removed. The glass is then to be inverted on the part. The application of single leeches to particular parts will be best done by means of a leech-glass, with or without the previous application of the feather. The less a leech is handled the better. The leeches being fixed, should be left alone, or covered with a napkin. Leeches sometimes remain for a considerable time dormant on their post. It is then recommended to sprinkle them with cold water, to hasten the sanguisuction. When it is wished to displace them, it may be done by a pin, or by a silk thread or horse hair drawn close between the leech and the skin. Dr. Price and some others aver, that the leech should not be *pulled* away, as in that case, the teeth of the animal will be left behind—the leech rendered incapable of again biting—and the wound be liable to fester. There are others, among them DURONDEAU, who believe that, when the leech has made his aperture and begun to suck, he withdraws his piercers—and, consequently, that Pliny's *story* of the death of Messalinus, from the forcible separation of leeches, is a *story* indeed.

The quantity of blood which the leech takes by suction is of comparatively trifling importance. Treble or quadruple the quantity is usually abstracted afterwards through the apertures. It is usual to sponge the bites with warm water, to encourage the bleeding. For our own parts, (and we have paid much attention to the subject,) we consider the best application to be a warm dry towel, removed from time to time, when it has become saturated with blood. This process saves a great deal of trouble, and we are convinced that the bites will bleed longer in this way than when they are irritated by the sponge. If the part can be conveniently immersed in a tepid bath, there can be no objection to this process. A poultice made with poppy-head decoction is a useful application after leeching, where it is desirable to soothe pain, as well as encourage bleeding. Cupping-glasses are sometimes applied over leech-bites, and where the parts will bear pressure, a great quantity of blood can thus be drawn, and with considerable rapidity. The glasses recommended by Mr. Welsh (suction by the mouth) may be useful at times. Another method of making the most of leeching, though at the expense of the leech's life, is to cut off its tail, and allow the current of blood to run through the animal. This process, however, often fails, by causing the leech to quit its hold. Dr. Price has known leeches survive this barbarous operation. To make the leech disgorge his cargo, people have been in the general habit of applying salt to their mouths—or even throwing them into a plate of salt. This is a cruel practice. The best way is to *strip* them, by laying hold of the tail, and drawing the leech backwards between two fingers pressed as close as possible together. This process, however, is not so easily done as people represent it. And so tenacious is the animal of its prey, that it often requires that degree of violence to empty it which injures its organization. It is proper to allow it to retain a portion (about a third) of the blood for its nutriment. Dr. Price says that the application of vinegar will make the leech disgorge the whole of the blood. We have tried it hundreds of times, and found it to fail more frequently than succeed.

The hæmorrhage which follows leeching, especially in the vascular skins of children, is sometimes troublesome, alarming—nay, we believe, fatal. Various means have been employed to stop the hæmorrhage. The branches of arteries are sometimes cut, and bleed profusely. The trunk of the temporal artery was opened by a leech in a young lad, and Sir Astley Cooper was obliged to cut down and divide the vessel completely, before the hæmorrhage could be arrested. A piece of adhesive plaster, with a compress and bandage, will generally stop the bleeding. But if the blood is still found to ooze beneath the dressings, they should be cast off, and the part well sprinkled with flower, gum arabic in powder, or what is best of all the puff-ball (Lycoperdon Bovista) over which pledgets of lint and the roller are again to be applied. A bit of felt, scraped from a beaver hat, is a good application, as is also a piece of sponge rolled in flower, starch, or gum arabic. Vinegar, ardent spirits, oil of turpentine, muriated tincture of iron, lunar caustic, are all powerful means of arresting the hæmorrhage. In an infant, where a single leech had made a large orifice on the sternum, all these means failed, and we were obliged to keep steady pressure with the finger on the wound for nearly three hours, before the bleeding would stop. Richerand has related the case of a child which was nearly exsanguined from leech-bleeding, and where nothing would stop the hæmorrhage till the actual cautery was applied.

In respect to the management of leeches after they have filled themselves, we are informed by Dr. Price that, in the course of numerous experiments, he has found that, if the leeches be permitted to retain the blood, and are merely thrown into a jar of fresh water, they will become remarkably healthy and firm in their texture; gradually losing their increased bulk, and, after the lapse of a month or two, capable of resuming their functions, as well as, or perhaps better than those which have been purged. He has, likewise, observed that leeches thus treated have remained healthy during a season of mortality among the others. The blood is preserved by the vital powers of their stomachs in a state of fluidity until it is entirely consumed.

But as leeches are sometimes quickly required after having been used, the best way is to *strip* them, and when wanted, they are to be immersed in porter, or coaxed by the application of the feathers, as before described.

We shall make no other apology for the length of this article than the following fact:—that in this metropolis alone, *four* importers of leeches receive about 150,000*l*. per month each, making a sum total of *seven millions two hundred thousand* in one year.

